# Reactive Oxygen Species: Friends and Foes of Signal Transduction

**DOI:** 10.1155/2012/534029

**Published:** 2012-03-11

**Authors:** Saverio Francesco Retta, Paola Chiarugi, Lorenza Trabalzini, Paolo Pinton, Alexey M. Belkin

**Affiliations:** ^1^Department of Clinical and Biological Sciences, University of Turin, Regione Gonzole 10, 10043 Orbassano, Italy; ^2^Department of Biochemical Sciences, University of Florence, viale Morgagni 50, 50134 Florence, Italy; ^3^Department of Biotechnology, University of Siena, Via Fiorentina 1, 53100 Siena, Italy; ^4^Department of Experimental and Diagnostic Medicine, University of Ferrara, Via Borsari 46, 44121 Ferrara, Italy; ^5^Department of Biochemistry and Molecular Biology, University of Maryland School of Medicine, 800 West Baltimore street, Baltimore, MD 21201, USA

The maintenance of highly regulated mechanisms to control intracellular levels of reactive oxygen species (ROS) is essential for normal cellular homeostasis. Indeed, most ROS, including free radicals and peroxides, are produced at low level by normal aerobic metabolism and play an important role in the redox-dependent regulation of many signaling processes. In contrast, excessive accumulation of ROS, resulting from an imbalance between ROS production and scavenging, leads to a condition of oxidative stress that can cause extensive oxidative damage to most cellular components, including proteins, lipids, and DNA, and may have pathophysiological consequences. Remarkably, oxidative stress has been clearly implicated in aging and the pathogenesis of several human diseases, including cardiovascular, metabolic, inflammatory, and neurodegenerative diseases and cancer. Thus, ROS may function as friends or foes of signal transduction depending on specific threshold levels and cell context.

To highlight the important topics in this evolving field the Journal of Signal Transduction presents a special issue on the involvement of ROS in physiological and pathological signal transduction processes from prokaryotes to low and high eukaryotes.

In particular, the topics covered in this special issue include ROS-mediated signaling in bacteria (in the first paper), the mechanisms by which ROS affect *Neurospora crassa* light signal transduction (in the second paper), the interplay between ROS and mitochondria in the control of cell death and aging (in the third and fourth papers) and cancer progression (in the fifth and sixth papers), the role of ROS in nuclear transport (in the seventh paper), the interplay between ROS and Ras GTPases (in the eighth paper), the role of ROS in the crosstalk between integrins and cadherins (in the ninth paper), integrin signaling (in the tenth paper), and skeletal muscle signaling (in the eleventh paper). These articles describe our current understanding of this field. Furthermore, this special issue highlights the importance of gaining a greater understanding of the physiological and pathological role of ROS in the perspective of defining new therapeutic strategies based on redox regulation of signal transduction processes.

*Saverio Francesco Retta* *Saverio Francesco Retta* *Paola Chiarugi* *Paola Chiarugi* *Lorenza Trabalzini* *Lorenza Trabalzini* *Paolo Pinton* *Paolo Pinton* *Alexey M. Belkin* *Alexey M. Belkin*

